# A phase 3, multicenter, randomized, double‐blind, vehicle‐controlled, parallel‐group study of 5% sofpironium bromide (BBI‐4000) gel in Japanese patients with primary axillary hyperhidrosis

**DOI:** 10.1111/1346-8138.15668

**Published:** 2021-01-07

**Authors:** Hiroo Yokozeki, Tomoko Fujimoto, Yoichiro Abe, Masaru Igarashi, Akiko Ishikoh, Tokuya Omi, Hiroki Kanda, Hiroto Kitahara, Miwako Kinoshita, Ichiro Nakasu, Naoko Hattori, Yuki Horiuchi, Ryuji Maruyama, Haruko Mizutani, Yoshiyuki Murakami, Chiharu Watanabe, Akihiro Kume, Takaaki Hanafusa, Masamitsu Hamaguchi, Akira Yoshioka, Yuriko Egami, Keizo Matsuo, Tomoko Matsuda, Motoki Akamatsu, Toshiyuki Yorozuya, Shinichi Takayama

**Affiliations:** ^1^ Department of Dermatology Tokyo Medical and Dental University Tokyo Japan; ^2^ Ikebukuro Nishiguchi Fukurou Dermatology Clinic Tokyo Japan; ^3^ Department of Pain Clinic NTT Medical Center Tokyo Tokyo Japan; ^4^ Igarashi Dermatology Clinic Tokyo Japan; ^5^ Kaminoge Hifuka Clinic Tokyo Japan; ^6^ Department of Dermatology Queen’s Square Medical Center Kanagawa Japan; ^7^ Mita Dermatology Clinic Tokyo Japan; ^8^ Kitahara Dermatology Clinic Tokyo Japan; ^9^ Kinoshita Dermatology Clinic Tokyo Japan; ^10^ Nemunoki Dermatology Clinic Kanagawa Japan; ^11^ Naoko Dermatology Clinic Tokyo Japan; ^12^ Akihabara Skin Clinic Tokyo Japan; ^13^ Maruyama Dermatology Clinic Tokyo Japan; ^14^ Mizutani Dermatology Clinic Tokyo Japan; ^15^ Mildix Skin Clinic Tokyo Japan; ^16^ Chiharu Dermatology Clinic Saitama Japan; ^17^ Dermatology and Ophthalmology Kume Clinic Osaka Japan; ^18^ Senri‐Chuo Hanafusa Dermatology Clinic Osaka Japan; ^19^ Hamaguchi Clinic Osaka Japan; ^20^ Yoshioka Dermatology Clinic Osaka Japan; ^21^ Ekihigashi Dermatology and Allergology Clinic Fukuoka Japan; ^22^ Matsuo Clinic Fukuoka Japan; ^23^ Tomoko Matsuda Dermatological Clinic Fukuoka Japan; ^24^ Kaken Pharmaceutical Co., Ltd. Tokyo Japan

**Keywords:** BBI‐4000, Hyperhidrosis Disease Severity Score, phase 3 study, primary axillary hyperhidrosis, sofpironium bromide gel

## Abstract

A phase 3 study was conducted to verify the efficacy and safety of 5% sofpironium bromide (BBI‐4000) gel (hereinafter referred to as sofpironium) administrated for 6 weeks in Japanese patients with primary axillary hyperhidrosis. The primary efficacy end‐point was the proportion of patients who satisfied both criteria of a Hyperhidrosis Disease Severity Score (HDSS) of 1 or 2 at the end of 6‐week treatment and a 50% or more reduction in total gravimetric weight of sweat at the end of treatment relative to baseline. A total of 281 patients were randomized to receive 5% sofpironium (141 patients) or vehicle (140 patients), and all patients were included in the full analysis set (FAS). In the FAS, 70.1% of patients were female, and the median age was 35.0 years. The proportion of patients who achieved the primary efficacy end‐point was 53.9% in the sofpironium group and 36.4% in the vehicle group, with a statistically significant difference of 17.5% (95% confidence interval, 6.02–28.93) between these two groups (*P* = 0.003). The incidence of adverse events was 44.0% in the sofpironium group and 30.7% in the vehicle group, and the incidence of adverse drug reactions was 16.3% in the sofpironium group and 5.0% in the vehicle group. Reported adverse events were generally mild or moderate in severity. In the sofpironium group, common events (incidence, ≥5%) were nasopharyngitis (14.2%) and dermatitis/erythema at the application site (8.5%/5.7%), with no serious adverse events reported. This study demonstrated the efficacy and safety of 5% sofpironium.

## INTRODUCTION

Hyperhidrosis, a skin disease involving excessive sweating beyond is physiologically required for thermoregulation, is clinically diagnosed when it results in emotional, physical or social distress and impairs patients’ quality of life (QOL).^1^ Hyperhidrosis can be primary or secondary. Approximately 93% of cases of hyperhidrosis are primary, and more than 90% of cases of primary hyperhidrosis are focal hyperhidrosis affecting the axillae, palms, soles and craniofacial areas.^1^ According to an epidemiological study conducted in Japan from 2009 to 2010 (5807 respondents), the prevalence of hyperhidrosis in Japan was 13.95% (810 persons), and 741 persons had primary focal hyperhidrosis. The prevalence of primary axillary hyperhidrosis was 5.75% (334 persons), with a mean onset age of 19.5 years. While 6.3% of patients with primary focal hyperhidrosis, including axillary hyperhidrosis, consulted a physician, 47.8% used non‐antiperspirant deodorants, highlighting the need for an appropriate treatment environment and education of patients.^2^

The Clinical Guidelines for Primary Focal Hyperhidrosis (2015 revised edition) published by the Japanese Dermatological Association recommend botulinum toxin type A and topical aluminum chloride for the treatment of primary axillary hyperhidrosis.^3^ Botulinum toxin type A, which was shown to be effective and safe in a double‐blind, placebo‐controlled, randomized study in Japanese patients with primary axillary hyperhidrosis,^4^ is a treatment for primary axillary hyperhidrosis with evidence of well‐controlled study. However, injection of high‐molecular‐weight botulinum toxin type A at 20–30 sites is painful,^5^ and use of botulinum toxin type A in primary axillary hyperhidrosis is covered by health insurance only in severe cases in Japan. On the other hand, topical aluminum chloride, which is the most common topical preparation for the treatment of hyperhidrosis,^5^ has not been studied in randomized controlled studies in primary axillary hyperhidrosis and is not covered by health insurance in Japan. Propantheline, an oral anticholinergic, is covered by health insurance for the treatment of hyperhidrosis in Japan, but one‐third of patients treated with oral anticholinergics are withdrawn from treatment because of adverse drug reactions.^5^ Taken together, there are unmet medical needs in the treatment of primary focal hyperhidrosis, and a treatment for primary focal hyperhidrosis that is safe and effective with evidence of well‐controlled study and is less likely to cause injection pain or systemic anticholinergic adverse drug reactions is necessary.

Human sweat glands are classified as eccrine or apocrine, and sweat resulting from hyperhidrosis is secreted by the eccrine sweat glands. The eccrine sweat glands are innervated by cholinergic nerves, and acetylcholine may stimulate postsynaptically localized M3 muscarinic receptors of the eccrine glands to induce sweating.^6^

Sofpironium bromide (BBI‐4000) is an M3 receptor ligand that resembles anticholinergic glycopyrronium bromide in chemical structure and has been developed as a retrometabolically designed drug that is chemically modified to contain ethyl ester.^7^, ^8^ A retrometabolically designed drug is a drug chemically modified to exert desired effects at the site of administration, after which it is quickly converted into inactive or less active metabolite(s) upon entry into the systemic circulation with an aim to reduce systemic adverse drug reactions. It has been reported that sofpironium bromide and (2*R*)‐BBI‐4000, which is a mixture of sofpironium bromide and its stereoisomer, were shown to be anticholinergic in a Magnus test using guinea pig ileum and an ocular administration study in rabbits, and that (2*R*)‐BBI‐4010, which is the de‐esterified form of (2*R*)‐BBI‐4000, is less anticholinergic than (2*R*)‐BBI‐4000.^9^ It is hypothesized that sofpironium bromide has a high binding affinity for the M3 acetylcholinergic receptor at the local site of administration, but is hydrolyzed at the ester linkage to less active metabolite(s) upon entry into blood. Based on this hypothesis, sofpironium bromide is expected to be effective in reducing sweating in primary axillary hyperhidrosis and to be associated with fewer systemic anticholinergic adverse drug reactions. We conducted a phase 3 study to verify the efficacy and safety of 5% sofpironium bromide gel (hereinafter referred to as sofpironium) administrated for 6 weeks in Japanese patients with primary axillary hyperhidrosis.

## METHODS

### Study design

A phase 3, multi‐center, randomized, double‐blind, vehicle‐controlled, parallel‐group study of 5% sofpironium was conducted in Japanese patients with primary axillary hyperhidrosis to verify the superiority of sofpironium applied to the axillae once daily at bedtime for 6 weeks over the vehicle in terms of efficacy based on the primary end‐point defined as the proportion of patients with a Hyperhidrosis Disease Severity Score (HDSS) of 1 or 2 at the end of treatment and a 50% or more reduction in total gravimetric weight of sweat at the end of treatment relative to baseline. The safety was also evaluated. This study was started after the protocol was approved by the institutional review board (IRB). The study was conducted in compliance with the protocol approved by the IRB, the principles of the Declaration of Helsinki, and the ministerial ordinance concerning Good Clinical Practice (Ordinance of the Ministry of Health and Welfare no. 28, 1997) and its revisions. Written informed consent was obtained from all patients before participation in the study. The study period was from 2018 to 2019 (JAPIC no. JapicCTI‐183948).

In this study, the assessment time points were baseline (three time points of baseline‐1, ‐2 and ‐3), week 2, week 4 and week 6 (three time points of week 6‐1, ‐2 and ‐3).

### Study patients

Patients who were aged 12 years or older at the time of informed consent and diagnosed with primary axillary hyperhidrosis meeting at least two of the following six conditions were eligible to participate in the study if all of the other inclusion criteria were met: (i) onset age of 25 years or younger; (ii) bilateral symmetrical sweating; (iii) no sweating during sleep; (iv) at least one episode of heavy sweating per week; (v) family history of axillary hyperhidrosis; and (vi) excessive sweating interfering with daily activities. The other four inclusion criteria were: (i) HDSS of 3 or 4 at baseline‐1 to ‐3; (ii) Hyperhidrosis Disease Severity Measure–Axillary (HDSM‐Ax) score of 2 or more at baseline‐1 to ‐3; (iii) gravimetric weight of sweat per side of 50 mg or more at two or more of baseline‐1 to ‐3; and (iv) subjective symptoms that had persisted for at least 6 months at the time of informed consent.

The main exclusion criteria were as follows: (i) secondary hyperhidrosis; (ii) heavy sweating triggered or worsened by menopause; (iii) thoracic sympathectomy was indicated; (iv) receiving or received treatment that might have affected the efficacy and/or safety evaluation in the study; and (v) concurrent or previous disease that might have affected the efficacy and/or safety evaluation in the study.

### Registration and assignment

Patients were registered by a registration center (EPS, Tokyo, Japan) independently. Patients were assigned to a sofpironium group or a vehicle group at a 1:1 ratio using the minimization method. The registration center sealed and securely retained allocation codes until database lock following the end of the study. Dynamic allocation based on sex, total gravimetric weight of sweat at baseline, HDSS at baseline and medical site was employed to balance the number of patients with regard to these factors that might have affected the efficacy evaluation.

### Treatment method

An adequate amount of 5% sofpironium or vehicle (vehicle alone) was thoroughly applied to both axillae once daily at bedtime for 6 weeks. The study drug was applied with an applicator to prevent the study drug exposure to a patient’s hand. In a preceding phase 2 dose‐finding study evaluating the efficacy and safety of sofpironium at concentrations of 5%, 10% and 15%, efficacy was observed at 5% and higher concentrations, whereas the incidence of systemic anticholinergic adverse drug reactions was lower in the 5% group than in the 10% and 15% groups. Hence, based on consideration of the balance between efficacy and safety, 5% sofpironium was recommended as the clinical dose.

### End‐points

The primary efficacy end‐point was the proportion of patients who satisfied both criteria with a HDSS^10^ of 1 or 2 at the end of 6‐week treatment and a 50% or more reduction in total gravimetric weight of sweat at the end of treatment relative to baseline. The gravimetric weight of sweat was measured as the difference in filter paper weight before and after 5 min of contact with the affected area. The primary efficacy end‐point was a combined end‐point composed of two outcomes, and each component of the primary end‐point was also evaluated as the secondary efficacy end‐point. The secondary efficacy end‐points were: (i) the proportion of patients with a HDSS of 1 or 2 at the end of treatment; (ii) the proportion of patients with a 50% or more reduction in total gravimetric weight of sweat at the end of treatment relative to baseline; (iii) the change in total gravimetric weight of sweat from baseline to the end of treatment; (iv) the change in Dermatology Life Quality Index (DLQI) (for axillary hyperhidrosis) score^11^ from baseline‐3 to week 6‐3; (v) the proportion of patients with an improvement of 1.5 or more in HDSM‐Ax score^12^ from baseline to the end of treatment; and (vi) the change in HDSM‐Ax score from baseline to the end of treatment.

The HDSS is used to assess the severity of primary focal hyperhidrosis by classifying subjective symptoms as follows: (i) sweating is never noticeable and never interferes with daily activities; (ii) sweating is tolerable, but sometimes interferes with daily activities; (iii) sweating is barely tolerable and frequently interferes with daily activities; and (iv) sweating is intolerable and always interferes with daily activities.

The HDSM‐Ax is a severity scale for axillary hyperhidrosis, and is a 5‐point (0–4) patient‐reported outcome measure with mean response scores comprised of 11 individual questions.^12^ The DLQI, which is designed to evaluate the skin disease‐related QOL, was modified into the DLQI (for axillary hyperhidrosis) to make it more suitable in assessment of primary axillary hyperhidrosis. Responses to 10 questions were scored and summed to obtain the DLQI (for axillary hyperhidrosis) score.

The gravimetric weight of sweat, HDSS and HDSM‐Ax score were measured at baseline‐1 to ‐3, week 2, week 4 and week 6‐1 to ‐3. The DLQI score was measured at baseline‐3, week 4 and week 6‐3.

The safety end‐points were adverse events, local tolerance (assessed by the physician and patients), vital signs and laboratory values (hematology, biochemistry and urinalysis). Adverse events were coded into the systemic organ class (SOC) and preferred term (PT) according to MedDRA/J version 21.1. An adverse event that, in the opinion of the sponsor with reference to that of the investigator, may be attributable to an anticholinergic effect was considered as an anticholinergic adverse event if the medical expert agreed. Multiple episodes of the same adverse event in the same patient were counted as one patient in calculation of the incidence. For local tolerance, the physician assessed dryness, erythema and scaling on a 5‐point sale (0, none; 1, minimal; 2, mild; 3, moderate; and 4, severe), and patients assessed burning sensation and itching on a 5‐point sale (0, none; 1, very mild; 2, mild; 3, moderate; and 4, severe).

### Statistical analysis

The target sample size was a total of 270 patients, consisting of 135 in the sofpironium group and 135 in the vehicle group based on the rationale described below. In a dose‐finding study in Japan, the proportion of patients with a HDSS of 1 or 2 at the end of treatment and a 50% or more reduction in total gravimetric weight of sweat at the end of treatment relative to baseline was 42.3% in the vehicle group and 63.5% in the sofpironium group. Assuming that the proportion in the present study would be 43% in the vehicle group and 63% in the sofpironium group, the necessary number of patients was therefore calculated to be 129 per group with a 1:1 ratio of vehicle versus sofpironium by χ^2^‐test with α = 0.05 and β = 0.1. Allowing for withdrawals, the target sample size was determined to be a total of 270 patients, with 135 patients per group.

Analyses were performed in accordance with a written procedure finalized before unblinding. The gravimetric weight of sweat, HDSS and HDSM‐Ax score at baseline and week 6 were measured at three time points each (baseline‐1 to ‐3 and week 6‐1 to ‐3, respectively), and the median of three measurements was used as data from each patient at baseline and week 6. For each end‐point, mean, standard deviation (SD), median, range, proportion (%) of patients and 95% confidence interval (CI) at each assessment time point were calculated for each treatment group for between‐group comparison. A χ^2^‐test was used for categorical variables, and an unpaired *t*‐test or ancova with baseline value as the covariate were used for quantitative variables. A two‐sided significance level of 5% was used, and no adjustment for multiplicity was made. SAS^®^ version 9.4 software (SAS Institute, Cary, NC, USA) was used for data analysis.

## RESULTS

### Study patients

**Figure 1:**
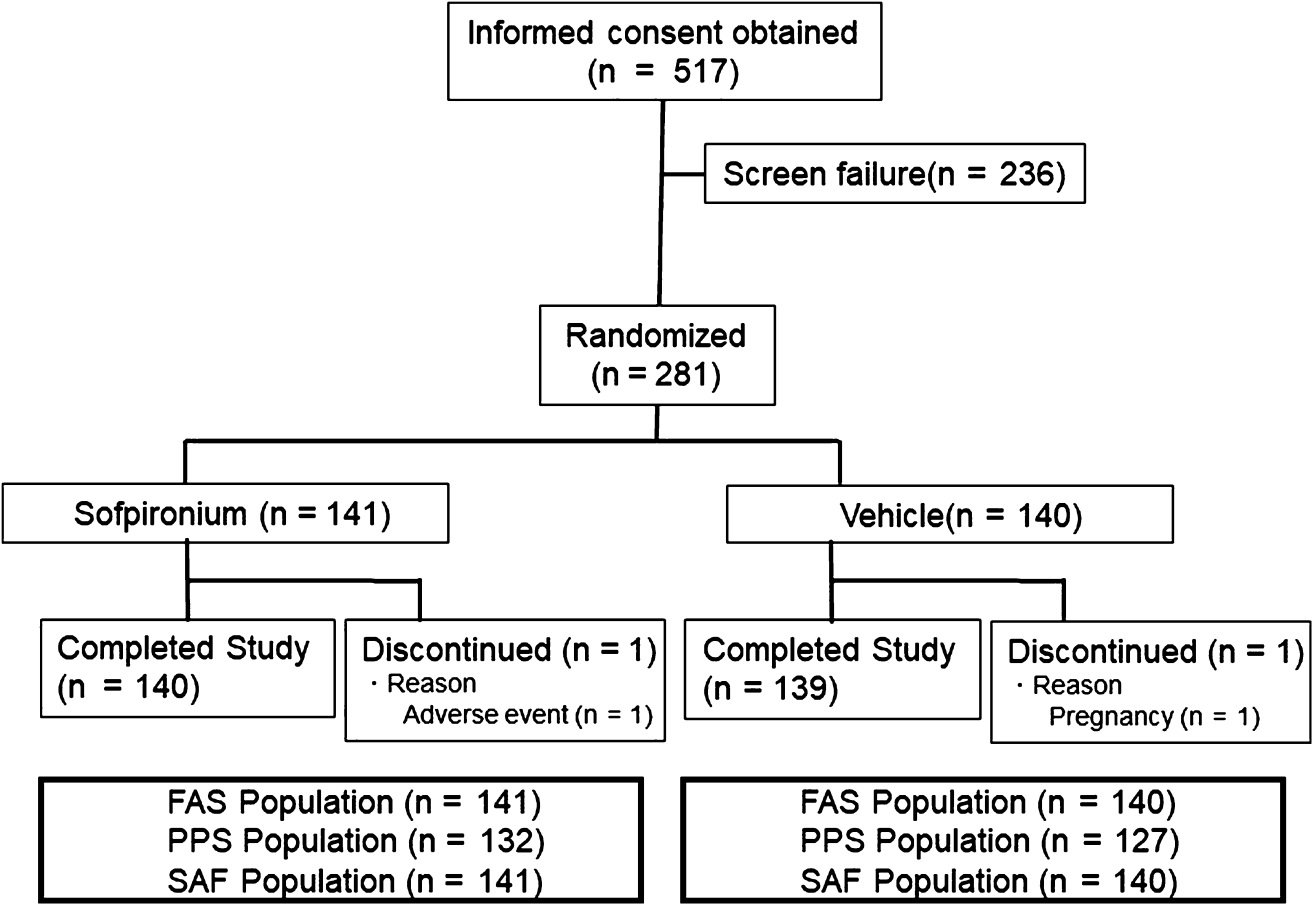
Patient disposition. FAS, full analysis set; PPS, per protocol set; SAF, safety analysis set.

The disposition of study patients is presented in Figure 1. Of the 517 Japanese patients with primary axillary hyperhidrosis who provided written informed consent, 281 were eligible and randomized to receive 5% sofpironium or vehicle (141 patients in the sofpironium group and 140 patients in the vehicle group). Study treatment was started in all of these 281 patients. Of the 281 patients who received study treatment, one in the sofpironium group and one in the vehicle group were discontinued from the study before week 6 due to an adverse event and pregnancy, respectively, and the remaining 279 patients completed the study at week 6.

All of the 281 randomized patients were included in the full analysis set (FAS), and 259 patients in the FAS, after excluding 22 patients, were included in the per protocol set (PPS). The reasons for excluding 22 patients were meeting any of the exclusion criteria, use of prohibited drug (or therapy), withdrawal from the study before efficacy assessment at week 6, deviation from the condition for measuring gravimetric weight of sweat at baseline or the end of treatment, end‐of‐treatment visit outside the acceptable range, study treatment compliance of less than 80% and failure to use the study drug on the day before the end‐of‐treatment visit. The safety analysis set (SAF) was identical to the FAS.

In the 281 patients of the FAS, there were more females than males, and the median age was 35.0 years. There were no imbalances in the patient baseline characteristics between the two treatment groups (Table 1).

**Table 1:** Baseline characteristics of study patients (FAS)

	Sofpironium (*n* = 141)	Vehicle (*n* = 140)	Total (*n* = 281)
Age (years)			
Mean (SD)	35.6 (13.44)	36.1 (12.44)	35.8 (12.93)
Median	35.0	36.0	35.0
Range	13–72	13–72	13–72
Sex (%)			
Male	43 (30.5)	41 (29.3)	84 (29.9)
Female	98 (69.5)	99 (70.7)	197 (70.1)
BMI (kg/m^2^)			
Mean (SD)	21.50 (3.292)	21.84 (3.043)	21.67 (3.170)
Median	20.96	21.36	21.22
Range	14.8–34.2	16.0–32.8	14.8–34.2
Total gravimetric weight of sweat (mg)			
Mean (SD)	228.0 (167.10)	226.3 (128.88)	227.1 (149.02)
Median	172.0	188.5	183.0
Range	105–1083	102–778	102–1083
HDSS, *n* (%)			
Grade 3	95 (67.4)	94 (67.1)	189 (67.3)
Grade 4	46 (32.6)	46 (32.9)	92 (32.7)
HDSM‐Ax score			
Mean (SD)	3.06 (0.514)	3.04 (0.572)	3.05 (0.543)
Median	3.09	3.00	3.09
Range	2.0–4.0	2.0–4.0	2.0–4.0
DLQI total score			
Mean (SD)	11.7 (4.96)	10.9 (4.42)	11.3 (4.71)
Median	11.0	11.0	11.0
Range	1–26	0–23	0–26

BMI, body mass index; DLQI, Dermatology Life Quality Index; HDSM‐Ax, Hyperhidrosis Disease Severity Measure–Axillary; HDSS, Hyperhidrosis Disease Severity Score; SD, standard deviation.

### Efficacy

#### Primary efficacy end‐point (FAS and PPS)

The efficacy was analyzed in the FAS as the primary analysis population, and also in the PPS.

In the FAS, the proportion of patients with a HDSS of 1 or 2 at the end of treatment and a 50% or more reduction in total gravimetric weight of sweat at the end of treatment relative to baseline was 53.9% in the sofpironium group and 36.4% in the vehicle group. A statistically significant difference of 17.5% (95% CI, 6.02–28.93) was observed between these two groups at this end‐point (*P* = 0.003). Similar results were observed in the PPS (Table 2).

**Table 2:** Summary of primary efficacy end‐point

Analysis population	Sofpironium	Vehicle	Difference, % (95% CI)	*P* *
	Achievement of primary efficacy end‐point, % (no. of patients)			
FAS	53.9% (76/141)	36.4% (51/140)	17.5% (6.02–28.93)	0.003
PPS	54.5% (72/132)	37.8% (48/127)	16.8% (4.78–28.72)	0.006

CI, confidence interval; FAS, full analysis set; PPS, per protocol set.

* χ^2^‐Test.

#### Secondary efficacy end‐points (FAS)

##### HDSS

**Figure 2:**
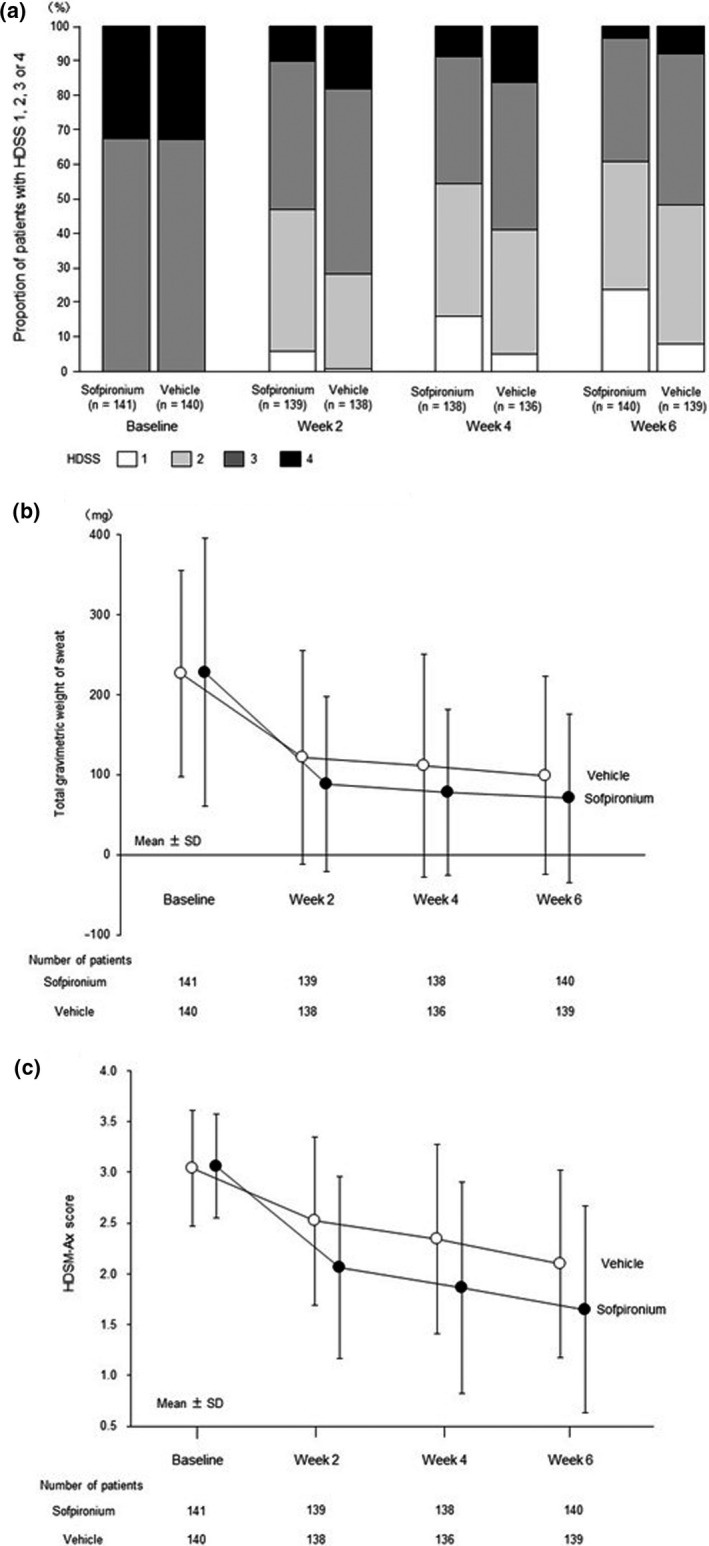
Changes in values of secondary efficacy end‐points at each evaluation point. (a) Changes in proportion of patients with Hyperhidrosis Disease Severity Score (HDSS) of 1, 2, 3 or 4. (b) Changes in total gravimetric weight of sweat. (c) Changes in Hyperhidrosis Disease Severity Measure–Axillary (HDSM‐Ax) score. Data from each patient at baseline and week 6 consisted of the median of measurements at three time points each (baseline‐1 to ‐3 and week 6‐1 to ‐3, respectively), and data from each patient at weeks 2 and 4 consisted of the measurement at one time point each. The patients with no data at each evaluation point were excluded from the analysis.

The proportion of patients with a HDSS of 1 or 2 at the end of treatment was 60.3% in the sofpironium group and 47.9% in the vehicle group (*P* = 0.036). The difference was 12.4% (95% CI, 0.86–23.99) between these two groups (Table 3). The proportion of patients with a HDSS of 1 or 2 was consistently higher from week 2 to 6 in the sofpironium group than in the vehicle group (Fig. 2a).

**Table 3:** Summary of secondary efficacy end‐points

Secondary efficacy end‐points	Sofpironium (*n* = 141)	Vehicle (*n* = 140)	*P*
Patients with HDSS 1 or 2 at week 6, *n* (%)	85 (60.3%)	67 (47.9%)	0.036*
Patients with ≥50% reduction in total gravimetric weight of sweat at week 6/at baseline, *n* (%)	109 (77.3%)	93 (66.4%)	0.042*
Mean change in total gravimetric weight of sweat from baseline to week 6 (SD)	–157.6 mg (149.32)	–127.6 mg (121.05)	－
Least squares mean	–157.1 mg	–128.1 mg	0.015^†^
Mean change in DLQI score from baseline to week 6 (SD)	–6.8 (4.94)	–4.5 (4.54)	<0.001^‡^
Patients with improvement ≥1.5 in HDSM‐Ax score from baseline to week 6, *n* (%)	68 (48.2%)	37 (26.4%)	<0.001*
Mean change in HDSM‐Ax score from baseline to week 6 (SD)	–1.41 (1.008)	–0.93 (0.902)	<0.001^‡^

BMI, body mass index; DLQI, Dermatology Life Quality Index; HDSM‐Ax, Hyperhidrosis Disease Severity Measure–Axillary; HDSS, Hyperhidrosis Disease Severity Score; SD, standard deviation.

* χ^2^‐Test.

^†^ ancova adjusting with baseline value.

^‡^ Unpaired *t*‐test.

##### Gravimetric weight of sweat

The proportion of patients with a 50% or more reduction in total gravimetric weight of sweat at the end of treatment relative to baseline was 77.3% in the sofpironium group and 66.4% in the vehicle group (*P* = 0.042). The difference was 10.9% (95% CI, 0.44–21.32) between these two groups (Table 3). The mean change in total gravimetric weight of sweat from baseline to the end of treatment was −157.6 mg in the sofpironium group and −127.6 mg in the vehicle group (*P* = 0.015), and the estimated between‐group difference in least squares mean was −28.9 mg (95% CI, −52.44 to −5.45) (Table 3). The mean total gravimetric weight of sweat was consistently lower from weeks 2 to 6 in the sofpironium group than in the vehicle group (Fig. 2b).

##### DLQI score

The mean change in DLQI score from baseline‐3 to week 6‐3 was −6.8 in the sofpironium group and −4.5 in the vehicle group (*P* < 0.001). The reduction was greater by −2.2 (95% CI, −3.36 to −1.13) in the sofpironium group than in the vehicle group (Table 3).

##### HDSM‐Ax score

The mean change in HDSM‐Ax score from baseline to the end of treatment was −1.41 in the sofpironium group and −0.93 in the vehicle group (*P* < 0.001). The reduction was greater by −0.48 (95% CI, −0.704 to −0.253) in the sofpironium group than in the vehicle group (Table 3). The proportion of patients with an improvement of 1.5 or more in HDSM‐Ax score from baseline to the end of treatment was 48.2% in the sofpironium group and 26.4% in the vehicle group (*P* < 0.001). The difference was 21.8% (95% CI, 10.78–32.82) between these two groups (Table 3). The mean HDSM‐Ax score was consistently lower from weeks 2 to 6 in the sofpironium group than in the vehicle group (Fig. 2c).

#### Analysis of efficacy in subgroups

Subgroup analyses stratified by age, sex, total gravimetric weight of sweat at baseline and HDSS at baseline, which were the factors that might have affected the efficacy evaluation, were performed. In subgroup analyses, improvement was consistently greater in the sofpironium group than in the vehicle group in all subgroups, as exemplified by a greater improvement in the sofpironium group than in the vehicle group in the subgroup of patients with severe disease (total gravimetric weight of sweat at baseline, ≥400 mg) (no data are presented).

### Safety

In this study, the safety was evaluated in 281 patients in the SAF. The incidence of adverse events was 44.0% (62/141 patients) in the sofpironium group and 30.7% (43/140 patients) in the vehicle group, and the incidence of adverse drug reactions was 16.3% (23/141 patients) in the sofpironium group and 5.0% (7/140 patients) in the vehicle group. By severity, adverse events were generally mild or moderate in both groups. In the sofpironium group, common events (incidence, ≥5%) were nasopharyngitis (14.2%, 20/141 patients), dermatitis at the application site (8.5%, 12/141 patients) and erythema at the application site (5.7%, 8/141 patients), all of which occurred more frequently in the sofpironium group than in the vehicle group (Table 4). Adverse drug reactions (PT) with an incidence of 2% or more in any treatment group were dermatitis at the application site (6.4% [9/141 patients] in the sofpironium group and 2.1% [3/140 patients] in the vehicle group), erythema at the application site (5.7% [8/141 patients] and 0% [0/140 patients]) and pruritus at the application site (2.1% [3/141 patients] and 0% [0/140 patients]). All of these events occurred at the application site of the study drug with a higher incidence in the sofpironium group than in the vehicle group.

**Table 4:** Incidence of treatment‐emergent adverse events (TEAE) in safety analysis set

	Sofpironium (*n* = 141)	Vehicle (*n* = 140)
	Incidence, *n* (%)	
TEAE		
Any	62 (44.0)	43 (30.7)
Drug‐related	23 (16.3)	7 (5.0)
Serious	0	2 (1.4)
Discontinuation due to TEAE	1 (0.7)	0
Death	0	0
TEAE by severity		
Mild	60 (42.6)	38 (27.1)
Moderate	2 (1.4)	4 (2.9)
Severe	0	1 (0.7)
Common TEAE reported in >2% of patients in sofpironium group		
Nasopharyngitis	20 (14.2)	8 (5.7)
Dermatitis at the application site	12 (8.5)	3 (2.1)
Erythema at the application site	8 (5.7)	1 (0.7)
Eczema	6 (4.3)	5 (3.6)
Miliaria	5 (3.5)	1 (0.7)
Acne	2 (1.4)	4 (2.9)
Tinea pedis	3 (2.1)	0
Pruritus at the application site	3 (2.1)	0
Eczema at the application site	3 (2.1)	1 (0.7)
Anticholinergic TEAE		
Thirst	2 (1.4)	0
Constipation	1 (0.7)	0
Mydriasis	1 (0.7)	0
Headache	0	1 (0.7)
Vision blurred	0	1 (0.7)

MedDRA version 21.1.

The incidence of adverse events at the application site of the study drug was 21.3% (30/141 patients) in the sofpironium group and 5.0% (7/140 patients) in the vehicle group, and the incidence of adverse drug reactions at the application site of the study drug was 15.6% (22/141 patients) in the sofpironium group and 3.6% (5/140 patients) in the vehicle group. These adverse events and adverse drug reactions occurred more frequently in the sofpironium group than in the vehicle group. By severity, all these events were mild, except for moderate urticaria in one patient in the sofpironium group.

In this study, there were no deaths. Other serious adverse events were diverticulitis and immunoglobulin A nephropathy in one patient each in the vehicle group, with none reported in the sofpironium group. The adverse event leading to withdrawal from the study was mild erythema at the application site in one patient in the sofpironium group, which was found to be resolving at a follow up after withdrawal. Anticholinergic adverse events were constipation, thirst and mydriasis in the sofpironium group, and blurred vision and headache in the vehicle group. All anticholinergic adverse events resolved without interruption or discontinuation of study treatment. Two adverse events led to interruption or discontinuation of study treatment in one patient in the sofpironium group (Table 4). Both events occurred at the application site (erythema and dermatitis at the application site) and were considered as adverse drug reactions. After interruption or discontinuation of study treatment, one resolved and the other was resolving.

For all variables of local tolerance at week 6, the median was 0 in both groups, and the mean was higher in the sofpironium group than in the vehicle group. There were no significant differences in vital signs between the treatment groups. For laboratory values over the study period and in individual patients, there were no notable changes in any parameters.

## DISCUSSION

In the 281 patients who participated in this study, the median age was 35.0 years and 70.1% of patients were female. Consistent with a finding from an epidemiological study in Japan that the age‐specific prevalence of primary focal hyperhidrosis was highest in the 25–29‐year category,^2^ relatively more patients aged 20–40 years were included in the present study, reflecting the age distribution of patients with primary axillary hyperhidrosis in Japan. While the epidemiological study reported a significantly higher prevalence of primary focal hyperhidrosis in males than in females,^2^ female patients with hyperhidrosis are more willing to receive treatment than male patients.^2^, ^13^ In the present study as well, female patients with primary axillary hyperhidrosis might have been more willing to participate in the study to receive a new treatment than male patients.

Patients with primary axillary hyperhidrosis who had a HDSS of 3 or 4 and a 5‐min gravimetric weight of sweat per side of 50 mg or more in both axillae at baseline were eligible to participate in this study. Given the similarity between the study patient population and the patient population that will use sofpironium after market launch, the efficacy and safety of sofpironium in the study may be generalizable to clinical practice. Primary axillary hyperhidrosis is a refractory disease that not only limits daily and social activities, but also causes psychological and emotional distress.^3^, ^14^, ^15^ In addition, this disease interferes with daily activities due to limited choice of clothing and frequent changes of clothes or showers;^16^ therefore, improvement to tolerable sweating with a HDSS of 1 or 2 after treatment is clinically significant. Furthermore, based on the belief that treatment‐induced inhibition of sweating can be evaluated objectively and quantitatively by assessing the gravimetric weight of sweat, the primary end‐point was defined as the proportion of patients with a HDSS of 1 or 2 at the end of treatment and a 50% or more reduction in total gravimetric weight of sweat at the end of treatment relative to baseline. In the FAS, which was the primary analysis population, the proportion of patients who achieved the primary end‐point was higher in the sofpironium group than in the vehicle group with a statistically significant difference, demonstrating the efficacy of sofpironium. Similar analysis results were observed in the PPS. The improvement in the vehicle group may be explained by the placebo effect in the axilla, a unique environment in which both emotional and thermoregulatory sweating occurs, that resulted in reduced emotional sweating and thus reduced gravimetric weight of sweat.

In addition, a greater improvement in the sofpironium group than in the vehicle group with a statistically significant difference was observed in all secondary end‐points. The secondary end‐points included each component of combined primary end‐point, and the results of the secondary end‐points consistently supported the efficacy as measured by the primary end‐point, and the changes over time indicated as early improvement as at week 2 in the sofpironium group. A phase 3, randomized, vehicle‐controlled studies of topical glycopyrronium, a topically administrated anticholinergic like sofpironium, was conducted in the USA and Germany. Topical glycopyrronium or vehicle was administrated to patients with primary axillary hyperhidrosis who had a HDSS of 3 or 4 for 4 weeks. Glycopyrronium significantly improved the patient assessment of sweating severity via the Axillary Sweating Daily Diary and the gravimetric weight of sweat as compared with the vehicle.^17^ These improvements in subjective symptoms and gravimetric weight of sweat achieved with glycopyrronium in patients with primary axillary hyperhidrosis may support the efficacy of sofpironium in the present study.

In subgroup analyses, improvement was consistently greater in the sofpironium group than in the vehicle group in all subgroups stratified by age, sex, gravimetric weight of sweat at baseline and HDSS at baseline. Sofpironium is therefore expected to be effective regardless of age, sex and disease severity.

In this study, the incidence of adverse events was 44.0% in the sofpironium group and 30.7% in the vehicle group, and reported adverse events were generally mild or moderate in severity, with no deaths reported in either group and no serious adverse events reported in the sofpironium group. The most common adverse event was nasopharyngitis, which occurred more frequently in the sofpironium group than in the vehicle group, but all events of nasopharyngitis in the sofpironium group were incidental cold symptoms and therefore considered unrelated to sofpironium treatment. The adverse event leading to withdrawal from the study was mild erythema at the application site in one patient in the sofpironium group, which was found to be resolving at a follow up after withdrawal. Adverse events due to pharmacological anticholinergic effects of sofpironium were thirst (1.4%, 2/141 patients), constipation (0.7%, 1/141 patients) and mydriasis (0.7%, 1/141 patients) in the sofpironium group, and all of these events were mild in severity and resolved without interruption or discontinuation of sofpironium, indicating that the safety risk due to anticholinergic effects of sofpironium is low and controllable. As mentioned in the Introduction section, one‐third of patients treated with an oral anticholinergic have been reported to withdraw from treatment because of anticholinergic adverse effects;^5^ therefore, the safety risk due to anticholinergic effects of sofpironium were expected to be lower than that of oral anticholinergics. The incidence of adverse events at the application site was 21.3% in the sofpironium group and 5.0% in the vehicle group, being higher in the sofpironium group than in the vehicle group. In the assessment of local tolerance, the mean was higher in the sofpironium group than in the vehicle group for all variables at week 6. In the sofpironium group, common adverse events (incidence) were dermatitis at the application site (8.5%), erythema at the application site (5.7%), miliaria (3.5%), pruritus at the application site (2.1%) and eczema at the application site (2.1%). Of these events, erythema at the application site in three patients and dermatitis at the application site in one patient led to interruption or discontinuation of sofpironium, and then resolved or were resolving. Taken together, sofpironium is associated with a risk of dermatitis and irritant reactions at the application site, but this risk is plausible in that sofpironium is intended for topical use and may be controllable because these changes were reversible and did not substantially preclude continued use. There were no notable changes in laboratory values or vital signs. In this study, the adverse events occurred in 30.7% in the vehicle group; however, the incidence is not higher than those of vehicle groups (32.3–56%) in the clinical studies of other topical preparations.^17^, ^18^, ^19^, ^20^, ^21^

This study had limitations: it was not designed to evaluate the long‐term safety and efficacy (a long‐term study is underway) or the efficacy and safety of sofpironium in combination with occlusive dressing therapy or other drugs.

This study demonstrated the efficacy of 5% sofpironium applied to the axillae once daily at bedtime for 6 weeks. Sofpironium is the first topical preparation in Japan that has been demonstrated to be effective and safe for treatment of primary axillary hyperhidrosis in a double‐blind, randomized, controlled study. Sofpironium is highly convenient in that it is a self‐administrated topical preparation, and this gel, unlike i.d. administrated botulinum toxin type A treatment, is not invasive. In addition, because the incidence of systemic adverse drug reactions of concern due to the nature of the anticholinergic was low, treatment with topical sofpironium may be less problematic than oral anticholinergic therapy. Dermatitis and irritant reactions at the application site occurred; however, many of these events were mild in severity, the incidences were not as high as expected as the topical preparation and treatment could be continued, indicating that the risk may be acceptable. In conclusion, 5% sofpironium was confirmed to be effective and safe in Japanese patients with primary axillary hyperhidrosis. It is expected to be widely used as first‐line treatment for this disease in Japan and improve the QOL of many patients.

## Conflict of Interest

This study was funded by Kaken Pharmaceutical. H. Y. received a consultancy fee and/or commission fee from Kaken Pharmaceutical. H. Y., T. F., M. I., T. O., H. K., R. M., Y. M., A. K. and A. Y. received fees as resource speakers from Kaken Pharmaceutical. H. Y. received fees for arranging education from Kaken Pharmaceutical. O. T. has stock in Kaken Pharmaceutical. M. A., T. Y. and S. T. are employees of Kaken Pharmaceutical and have stock in Kaken Pharmaceutical. With funding from Kaken Pharmaceutical, Medical Professional Relations assisted in the writing and editing of this paper.

## References

[1] Nawrocki S , Cha J . The etiology, diagnosis, and management of hyperhidrosis: A comprehensive review: Etiology and clinical work‐up. J Am Acad Dermatol 2019; 81: 657–666. 30710604 10.1016/j.jaad.2018.12.071

[2] Fujimoto T , Kawahara K , Yokozeki H . Epidemiological study and considerations of focal hyperhidrosis in Japan. From questionnaire analysis. J Dermatol 2013; 40: 886–890. 24106874 10.1111/1346-8138.12258

[3] Fujimoto T , Yokozeki H , Katayama I *et al.* Guidelines of the Japanese dermatological association, clinical guidelines for primary focal hyperhidrosis (revised edition in 2015). Jpn J Dermatol 2015; 125: 1379–1400. (article in Japanese).

[4] Ohshima Y , Tamada Y , Yokozeki H *et al.* The efficacy and safety of botulinum toxin type a in patients with primary axillary hyperhidrosis. Nishinihon. J Dermatology 2013; 75: 357–364. (article in Japanese).

[5] Nawrocki S , Cha J . The etiology, diagnosis, and management of hyperhidrosis: a comprehensive review: therapeutic options. J Am Acad Dermatol 2019; 81: 669–680. 30710603 10.1016/j.jaad.2018.11.066

[6] Schlereth T , Dieterich M , Birklein F . Hyperhidrosis ‐ causes and treatment of enhanced sweating. Dtsch Arztebl Int 2009; 106: 32–37. 19564960 10.3238/arztebl.2009.0032 PMC2695293

[7] Lee HJ , Soliman MR . Anti‐inflammatory steroids without pituitary‐adrenal suppression. Science 1982; 215: 989–991. 6296999 10.1126/science.6296999

[8] Bodor N , Sloan KB , Higuchi T , Sasahara K . Improved delivery through biological membranes. 4. Prodrugs of L‐dopa. J Med Chem 1977; 20: 1435–1445. 915903 10.1021/jm00221a014

[9] Wu WM , Buchwald P , Mori N , Ji F , Wu J , Bodor N . Pharmacokinetic and pharmacodynamic evaluations of the zwitterionic metabolite of a new series of N‐substituted soft anticholinergics. Pharm Res 2005; 22: 2035–2044. 16170596 10.1007/s11095-005-8174-z

[10] Solish N , Bertucci V , Dansereau A *et al.* A comprehensive approach to the recognition, diagnosis, and severity‐based treatment of focal hyperhidrosis: recommendations of the Canadian Hyperhidrosis Advisory Committee. Dermatol Surg 2007; 33: 908–923. 17661933 10.1111/j.1524-4725.2007.33192.x

[11] Finlay AY , Khan GK . Dermatology Life Quality Index (DLQI)–a simple practical measure for routine clinical use. Clin Exp Dermatol 1994; 19: 210–216. 8033378 10.1111/j.1365-2230.1994.tb01167.x

[12] Kirsch BM , Burke L , Hobart J , Angulo D , Walker PS . The hyperhidrosis disease severity measure‐axillary: conceptualization and development of item content. J Drugs Dermatol 2018; 17: 707–714. 30005091

[13] Sammons JE , Khachemoune A . Axillary hyperhidrosis: a focused review. J Dermatolog Treat 2017; 28: 582–590. 28318360 10.1080/09546634.2017.1309347

[14] Strutton DR , Kowalski JW , Glaser DA , Stang PE . US prevalence of hyperhidrosis and impact on individuals with axillary hyperhidrosis: results from a national survey. J Am Acad Dermatol 2004; 51: 241–248. 15280843 10.1016/j.jaad.2003.12.040

[15] Hornberger J , Grimes K , Naumann M *et al.* Recognition, diagnosis, and treatment of primary focal hyperhidrosis. J Am Acad Dermatol 2004; 51: 274–286. 15280848 10.1016/j.jaad.2003.12.029

[16] Hamm H . Impact of hyperhidrosis on quality of life and its assessment. Dermatol Clin 2014; 32: 467–476. 25152339 10.1016/j.det.2014.06.004

[17] Glaser DA , Hebert AA , Nast A *et al.* Topical glycopyrronium tosylate for the treatment of primary axillary hyperhidrosis: Results from the ATMOS‐1 and ATMOS‐2 phase 3 randomized controlled trials. J Am Acad Dermatol 2019; 80: 128–138. 30003988 10.1016/j.jaad.2018.07.002

[18] Saeki H , Baba N , Oshiden K *et al.* Phase 2, randomized, double‐blind, placebo‐controlled, 4‐week study to evaluate the safety and efficacy of OPA‐ 15406 (difamilast), a new topical selective phosphodiesterase type‐4 inhibitor, in Japanese pediatric patients aged 2–14 years with atopic dermatitis. J Dermatol 2020; 47: 17–24. 31713267 10.1111/1346-8138.15137 PMC6972691

[19] Nakagawa H , Nemoto O , Igarashi A *et al.* Phase 2 clinical study of delgocitinib ointment in pediatric patients with atopic dermatitis. J Allergy Clin Immunol 2019; 144: 1575–1583. 31425780 10.1016/j.jaci.2019.08.004

[20] Bissonnette R , Papp KA , Poulin Y *et al.* Topical tofacitinib for atopic dermatitis: a phase IIa randomized trial. Br J Dermatol 2016; 175: 902–911. 27423107 10.1111/bjd.14871

[21] Warshaw EM , Wohlhuter RJ , Liu A *et al.* Results of a randomized, double‐blind, vehicle‐controlled efficacy trial of pimecrolimus cream 1% for the treatment of moderate to severe facial seborrheic dermatitis. J Am Acad Dermatol 2007; 57: 257–264. 17188780 10.1016/j.jaad.2006.11.007

